# Learning Type I and Type II regularities between multiple sequentially presented stimulus categories

**DOI:** 10.1007/s00426-025-02180-7

**Published:** 2025-09-18

**Authors:** Vedant Biren Shah, René Schlegelmilch, Bettina von Helversen

**Affiliations:** Allgemeine Psychologie, University of Bremen, Bremen, Germany

## Abstract

Classification is a common cognitive task, which requires assigning objects or events to categories based on shared features or rules (e.g., red objects are fruit, brown objects are mushrooms). In everyday scenarios, however, objects usually belong to more than one category (e.g., red objects can also be classified as edible, and brown objects could be poisonous). This study investigates whether humans can learn corresponding regularities between outcomes of such multiple categorizations when performed in a series of decisions for each stimulus. We therefore translated classical category learning designs, known as Type I (one-dimensional rule) and Type II (disjunctive rule), into a temporal context. We compared these cases to conditions in which no correlations existed between the series of categorization outcomes, and only the visual stimulus predicted each category outcome. Besides the structural complexity, we also tested in Type I scenarios whether learning and generalization were moderated by the temporal proximity of the successive decisions (adjacent vs. non-adjacent categorizations). The results show that participants can abstract away from the visual stimulus with a temporal Type I regularity, but there was no evidence for a corresponding effect with a temporal Type II regularity. The role of adjacency was not clear-cut, but there was no strong evidence favoring stronger performance with adjacent relative to non-adjacent categorizations. We discuss these findings before the background of category- and artificial grammar-learning research, and expand on potential moderating factors such as the cognitive effort of keeping the necessary amount of information in working memory and the modality of category predictors when determining whether people will extract rules or rely on memory-based learning.

**Supplementary Information:**

The online version contains supplementary material available at 10.1007/s00426-025-02180-7.

## Introduction

Patterns are fundamental to human learning and cognition, from recognizing that “red things are likely fruit” to understanding that “been” often follows “has”. Cognitive scientists have explored these pattern-learning processes from various angles, typically employing trial-by-trial observation of stimulus pairs. This includes learnable temporal regularities such that one stimulus predicts the next stimulus in time, which could be syllables or symbolic elements predicting each other, as studied in artificial grammar-learning (e.g., Gomez, 2002; Newport et al., 2004; Romberg & Saffran, 2013; Lu & Mintz, 2021; Vuong et al., 2016; Iao et al., 2021), or visual stimuli predicting subsequent outcomes or labels (e.g., a small, green stimulus predicts the category vegetables), as studied in category learning (e.g., Shepard et al., 1961; Pothos & Wil, 2011). These two types of relationships often co-occur in everyday life, but their integration has received little attention in research. For example, one might learn to categorize a “red” stimulus as a “fruit” (e.g., fruit vs. mushroom) and also as “edible” (e.g., edible vs. poisonous), with the two categorization decisions occurring sequentially for the same stimulus. In such cases, not only can the stimulus (e.g., “red”) predict both outcomes, but the outcome of the first task (e.g., “fruit”) could also help predict the second (e.g., “edible”). Thus, in this case, a relationship exists between the outcomes of the categorization tasks where the result of one decision reliably predicts the next, independently of the initial stimulus. We refer to such relationships between outcomes of sequential categorization tasks as temporal regularities. In the present work, we investigate whether—and under what conditions—people are able to abstract such temporal regularities and whether they can use them to support generalization for novel stimuli. Across two studies, we explore how learning of temporal regularities is influenced by (1) the temporal proximity between decisions—that is, whether the predictive relationships involve immediately successive or more temporally separated steps (Study 1), and (2) the complexity of the relationships (Study 2).

For this, we extend a classical visual category-learning design (Shepard et al., 1961) by incorporating temporal regularities that link multiple category decisions. That is, whereas traditional designs present all stimulus features simultaneously as to-be-classified exemplar, we enrich the single-exemplar episodes by multiple inter-related classifications in a sequence. For clarity, we use the term “sequence” to refer to multiple decisions for the same stimulus as “within-exemplar” temporal regularities, unlike research on exemplar “sequencing” (or grouping) of exemplars (e.g., Carvalho, 2014; Carvalho & Goldstone, 2015; Sorensen & Woltz, 2016; Abel et al., 2021a) highlighted in the next section. The current approach is closer to causal learning research (see Lagnado et al., 2007; Kim & Rehder, 2011; Rehder & Murphy, 2003; Sloman & Lagnado, 2015), which, however, focuses on the influences of prior knowledge, while our design uses artificial stimuli and classifications to isolate basic learning mechanisms, minimizing such influences.

We therefore translate well-established artificial visual structure types, known as Type I, II, and VI (Shepard et al., 1961), into analogous temporal-regularity structures between successive decisions. Specifically, Type VI acts as a baseline condition with no temporal regularity, Type I involves a simple predictive relationship where the outcome of one decision task consistently forecasts another (Study 1), and Type II requires the integration of information from two preceding classification outcomes (Study 2). Our primary objective is to investigate whether the mechanisms underlying learning of temporal regularities parallel those found in classical visual category learning (e.g., Shepard et al., 1961; Nosofsky et al., 1994b; Lewandowsky, 2011; Schlegelmilch et al., 2022; Kruschke, 1992; Kurtz et al., 2013). The subsequent sections elaborate on this conceptual framework and further develop our hypotheses by drawing on insights from both category-learning and artificial grammar-learning research (e.g., Wilson et al., 2020; Pothos & Wills, 2011) on the roles of adjacency and complexity.

### Visual category learning: learning Type I, Type II and Type VI structures

**Fig. 1:**
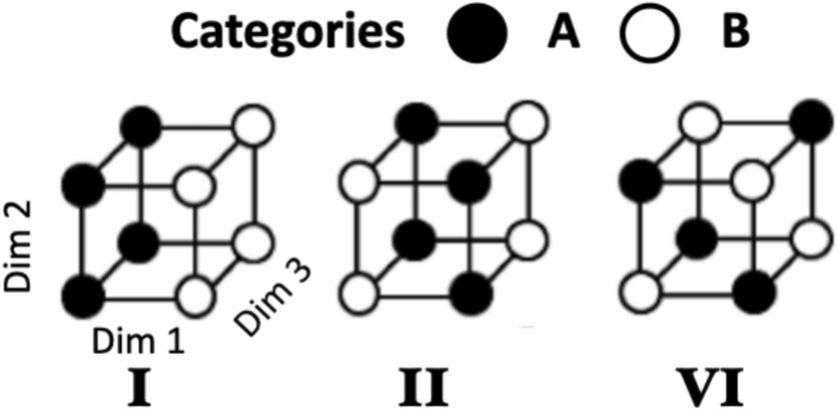
Category Structures. Type I, II, and VI from Shepard et al. (1961). Coordinates reflect three binary dimensions (e.g., Dim 1 = size: large vs. small). Shading reflects the assigned category (A vs. B). See further text

A classic paradigm in visual category learning, introduced by Shepard et al. (1961), investigates how the complexity of six different category structures affects learning performance (see also Nosofsky et al., 1994a; Love, 2002; Kurtz et al., 2013; Mathy & Bradmetz, 2011). Each of these six problems contains eight stimuli with three binary features (e.g., size [small vs. large], shape [circle vs. square], shading [black vs. white]) but differing in how they predict the stimulus outcomes. This paper focuses on three structures: Type I, Type II, and Type VI. Type I problem are one-dimensional and can be solved by noticing that the size feature perfectly predicts the two categories (small$\rightarrow$A and large$\rightarrow$B). Type II problems (Shepard et al., 1961), also commonly known as Exclusive-Or (XOR), can be solved by a combination of two features (e.g., size [small vs. large], shape [triangle vs. square]). In contrast, Type VI problems are unstructured, such that solving the problem requires memorizing every stimulus and its category (e.g., stimulus S$_1$: small-black-square$\rightarrow$A), illustrated in Fig. 1.

Research employing these and similar tasks has been argued to dissociate processes of abstracting rules from those of learning (idiosyncratic) memory representations (e.g., Nosofsky et al., 1994b; Schlegelmilch et al., 2022; Kurtz et al., 2013). Abstraction refers to a cognitive process of recognizing that one feature predicts the category membership of the stimulus in Type I and two features disjunctively predict the category membership in Type II, while any other feature could be ignored, such that both solutions come with a notion of rule-based learning. In comparison to Type I, however, the Type II (XOR) problem is of higher complexity, forming a disjunctive category structure (see Fig. 1). Accordingly, when such relations do not exist but only idiosyncratic perceptual features of each stimulus predict the category membership (i.e., feature-outcome combination), as in Type VI, it is assumed that people switch to a memory-based learning process (i.e., storing category exemplars). In this vein, people generally solve Type I tasks more quickly than Type II tasks. However, both Type I and Type II tasks are learned more quickly than Type VI tasks, suggesting that people can discover these systematic relations, which speeds up learning and improves performance (e.g., Shepard et al., 1961; Nosofsky et al., 1994a; Lewandowsky, 2011). Here, we aim to understand whether the same learning patterns can be found when Type I and Type II structures are implemented in inter-category association in sequentially occurring tasks. For example, consider two classification tasks that are performed sequentially, with each task requiring categorizing the stimulus in one of two categories. In a Type I structure, the outcome of the first categorization task would predict the outcome in the subsequent categorization task 2. In a Type II structure, a temporal regularity would require two categorization outcomes (from task 1 and task 2) together to predict the outcome of the third categorization task, detailed later.

In sum, while we extend a classic design to include multiple sequential categorization decisions (within exemplar episode), note that previous research has examined how the sequence of stimulus presentation—such as interleaved versus blocked exemplars—can affect category learning and long-term retention (between-exemplar episodes; e.g., Brunmair & Richter, 2019; Carvalho, 2014; Carvalho & Goldstone, 2015; Abel et al., 2021b; Abel et al., 2021a). These investigations focus on grouping effects across stimuli rather than the temporal structure within a single exemplar that are the focus of our research. Our perspective invites connections with research on temporal pattern learning in domains such as artificial grammar learning and the serial response time paradigm, where sequence effects have been widely studied. In the following section, we review insights from these fields of research focusing on factors that may influence learning temporal Type I regularities and using them for generalization.

### Temporal regularity: visual and verbal elements

Some insights into learning of temporal regularities stem from studies on artificial grammar learning and the serial response time paradigm. In these studies, participants observe sequences of grammatical elements in quick succession, such as syllables, sounds, or visual shapes, following specific sequential or transitional patterns (e.g., D [dee] follows B [bee] with 100% or 80% transitional probability). Knowledge acquisition of these temporal regularities is typically assessed through grammatical judgments, where participants evaluate novel sequences as “grammatical” based on their similarity to observed patterns, or through reproduction tasks, where participants recreate observed sequences using assigned keyboard buttons. Response times in the latter are measured to assess learning benefits compared to random sequences. Research has demonstrated that humans can learn such regularities through observation, regardless of explicit instructions to attend to sequential order (for an overview see Wilson et al., 2020). Typical research questions concern the role of implicit (unaware) versus explicit (aware) learning of regularities (for an overview see Frensch & Rünger, 2003), and the factors that influence how well regularities are learned (see Wilson et al., 2020).

One factor suggested as being relevant in temporal regularity in category learning also concerns categorization, which is the temporal proximity or adjacency of observed elements in a sequence. Saffran et al. (1996) investigated participants’ ability to learn regularities between directly neighboring (adjacent) speech sounds, presenting trisyllabic words with regularities set between adjacent syllables (e.g., ba$\rightarrow$da and pa$\rightarrow$do in a stream of ba-da-ku, pa-do-ti, ba-da-ti, etc.). Participants accurately recognized these regularities when differentiating between words (see also Gomez, 2002; Romberg & Saffran, 2013; Deocampo et al., 2019; Newport et al., 2004). However, researchers in artificial grammar-learning often posit that learning performance declines with the increased complexity of regularities (Wilson et al., 2020), including non-adjacent regularities where two elements appear with intervening elements between them (e.g., ba$\rightarrow$te when observing ba-di-te, ba-ku-te, or gu$\rightarrow$do when observing gu-di-do, gu-ku-do, etc.; see Gomez, 2002; Newport et al., 2004; Chen & Cat, 2015; Fitch & Hauser, 2004; Gebhart et al., 2009; Reber, 1967), while some studies investigating concurrent adjacent and non-adjacent regularities indicate that both are learned (e.g., Deocampo et al., 2019; Conway et al., 2020). Notably, adjacent regularities can be learned without explicit instructions to pay attention to them when reading a sequence of digits or syllables, which does not seem to be the case for non-adjacent regularities (Pacton et al., 2015). Here, we investigate whether similar effects are observed when participants learn sequentially presented categorization tasks, and whether the complexity of the regularity (adjacent vs. non-adjacent temporal regularities) has any effect.

Additionally, artificial-grammar research focuses on the influence of transitional or conditional probability on learning, which describes how accurately a subsequent element can be predicted based on previously experienced elements, such as 70% versus 90% transitional probability (also termed as statistical learning, e.g., Hunt & Aslin, 2002; Stadler, 1992; Poletiek & Wolters, 2009; Fiser & Aslin, 2002; Marcovitch & Lewkowicz, 2009). Researchers investigate two types of transitions: first-order and second-order transitional probabilities, which differ in complexity. First-order transitions can be equated to the previously described Type I category structure, while the Type II category structure is part of the class of second-order transitions. Research investigating learning of transitional probabilities in sequences of symbolic elements shows evidence that human and non-human animals can learn first- and second-order transitions (Rey et al., 2022; Lazartigues et al., 2022). However, learning second-order transitions generally requires more extensive training than first-order transitions.

In this vein, Lazartigues et al. (2022) found evidence for learning a Type I structure but relatively weak evidence of learning a Type II structure in a serial response-time task. Participants were shown red dots on the screen, with the possibility that the dots could appear in one of nine positions. The dots appeared in a sequence of three, and participants had to click on the dots as quickly as possible after they appeared on the screen. The authors varied regularities within the sequence, comparing structures with adjacent and non-adjacent first-order transitions (i.e., in the adjacent condition, the first dot predicted the location of the second dot, and in the non-adjacent, the first dot predicted the third dot location) and second-order transitions (XOR; i.e., the position of the first two dots combined predicts the location of the third dot). During acquisition, there was faster learning in the condition with adjacent first-order transitions compared to the conditions with non-adjacent first-order transitions or second-order transitions. However, in a subsequent switch phase where the predictable element was exchanged for an unpredictable one, they observed increased response times to the unpredictable element in both non-adjacent and XOR conditions, indicating some degree of regularity-based interference. In summary, people readily acquire temporal regularities in letter or visual stimulus sequences, particularly when first-order transitions occur on directly adjacent elements (see also Wilson et al., 2020; Newport et al., 2004; Lazartigues et al., 2022) This acquisition is more pronounced compared to other regularities such as non-adjacent regularities or more complex second-order XOR structures (Lazartigues et al., 2022; Hunt & Aslin, 2002; Wilson et al., 2020).

## Overview of the present research and predictions

The current research integrates concepts from category learning and temporal regularity acquisition to examine whether humans can utilize temporal regularities between multiple categorical outcomes that are carried out in a sequence. We focus on scenarios in which several idiosyncratic stimuli (S1, S2, ..., SN) are each paired with three tasks within a temporal episode: Upon presentation of S1, the first task is to classify it into one of two categories (e.g., “A” or “B”). After receiving feedback, the second categorization task follows, and the same stimulus needs to be classified into two further categories (e.g., “C” or “D”). Afterwards, a third categorization task follows, and so on. We denote the sequence of three decisions as S $\rightarrow$ O$^1$ $\rightarrow$ O$^2$ $\rightarrow$ O$^3$,(with O$^1$, O$^2$, O$^3$, denoting the outcomes of task 1, 2, and 3). The participant’s task is to predict the category outcomes (O) for each task based on the same stimulus (S). Study 1 investigates the potential benefits of a Type I structure embedded in sequential outcomes and explores how the adjacency of these regularities affects learning. Study 2 extends this inquiry by examining whether learning advantages persist in the presence of a more complex temporal Type II conditional regularity. We aim to enhance our understanding of human learning capabilities by investigating these cognitive processes in structured, sequential decision-making contexts.

### Using temporal regularity in category learning: Type I structure

**Fig. 2:**
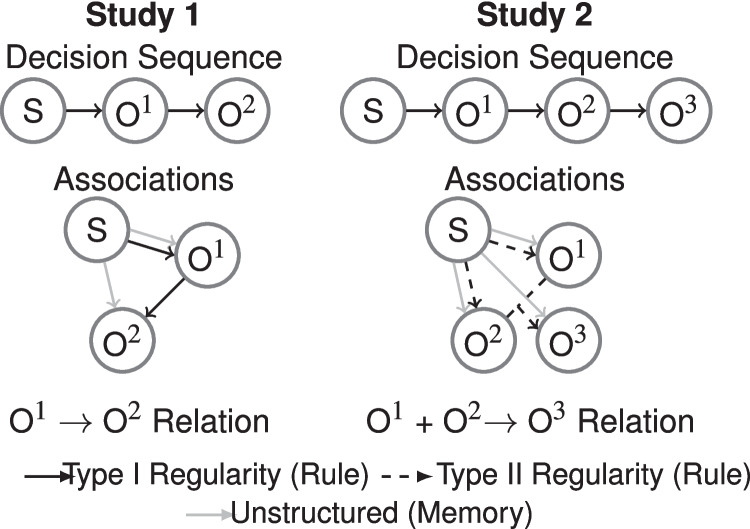
Schematic of Decision Sequence and Associations between Stimuli and Category Outcomes. S=Stimulus, O$^1$ = category feedback of first decision (categories A vs. B), O$^2$ = category feedback of second decision (categories C vs. D). See further text

**Fig. 3:**
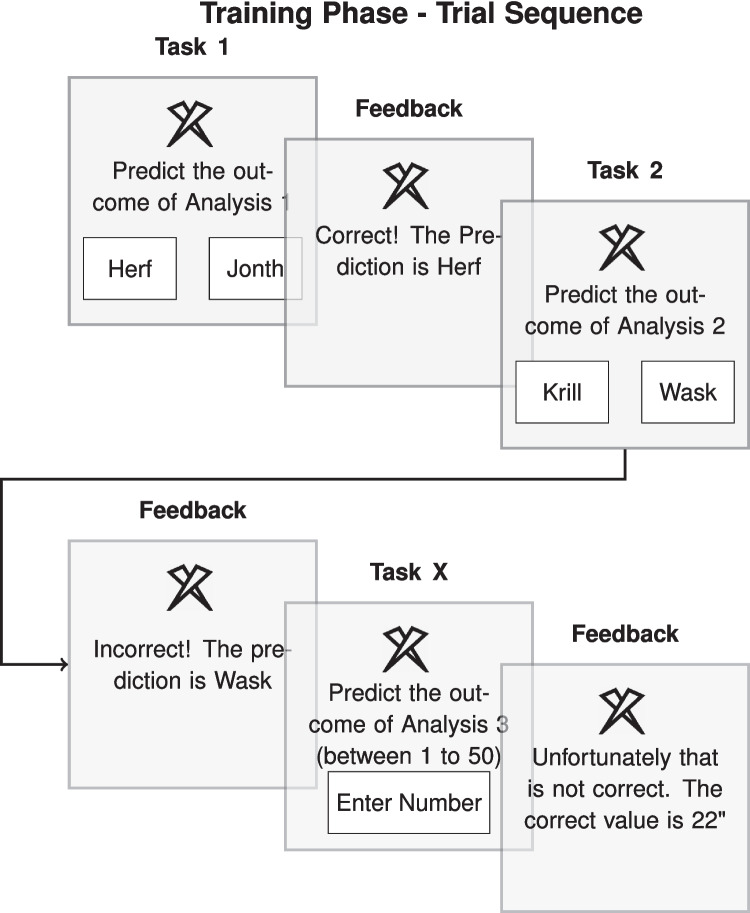
Example Trial Procedure. Sequence of tasks consisting of two categorization tasks (task 1 and task 2) and one estimation task (task X). Task 1 and task 2 have binary outcomes with O$^1$: Herf versus Jonth and O$^2$: Krill versus Wask respectively

Study 1 examines the simplest case where a stimulus has a sequence of three tasks: two categorization tasks and one estimation task. Figure 3 illustrates an example of a single complete trial where the stimulus (S) is present while performing each task. Task 1 and task 2 have binary outcomes with O$^1$: Herf versus Jonth and O$^2$: Krill versus Wask, respectively. The final task (X) is an estimation task with a range between 1 and 50. This sequence is formally denoted as S$\rightarrow$O$^1\rightarrow$O$^2\rightarrow$X as in Fig. 2. As each stimulus is categorized into two categories, regularity is defined as: outcome “Herf” in O$^1$ predicts “Krill” in O$^2$ (Herf$\rightarrow$ Krill), and outcome “Jonth” predicts outcome “Wask” (Jonth $\rightarrow$ Wask) (these stimulus-outcomes are counterbalanced between participants). This corresponds to a Type I structure with a single predictive binary dimension, where the outcome of the first task perfectly predicts category membership in the second task. The design employs idiosyncratic stimuli (S) that preclude the identification of visual feature dimensions for predicting category membership but allow for direct stimulus-outcome associations. In the Type I condition, participants can learn either through memorization of stimulus-outcome associations or by recognizing the category-category association. We compare this to a control condition (Type VI) where only stimulus-outcome associations can solve the task. If participants fail to abstract the Type I regularity, performance should not differ from the control condition. Figure 2 (left) illustrates these two alternative solutions: (1) associating each stimulus with both sequential categories (memorization), (2) making the second category contingent on the first (Type I rule learning).

Drawing on established category-learning research (e.g., Shepard et al., 1961; Nosofsky et al., 1994a; Lewandowsky, 2011), we anticipate significantly faster learning of Type I structures compared to Type VI. A second key feature of our design is that it allows for testing the generalization of learned regularities to novel stimuli, which is central in our design to determine whether abstraction took place. After the initial learning phase, the procedure therefore presents new stimuli with feedback only for the first task (O$^1$). Thus, even without knowing the exact stimulus, the participants could use the feedback of the O$^1$ task to accurately predict O$^2$. We hypothesize that if participants successfully abstract the rule, their behavior should be consistent with it for novel stimuli in this generalization test phase.

Informed by artificial grammar-learning research, we further examine whether learning performance and generalization are influenced by the adjacency of related categorization tasks. To this end, we introduced a third task X, and compared two conditions: one in which the third task appears at the end of the decision sequence, S$\rightarrow$O$^1\rightarrow$O$^2\rightarrow$X, and one where task X intervenes between the two related tasks, S$\rightarrow$O$^1\rightarrow$X$\rightarrow$O$^2$. We refer to the former as “adjacent” condition, and to the latter as “non-adjacent” reflecting whether the two related tasks O$^1$ and O$^2$ are, respectively, close versus distant from each other. Note, in task X, participants estimated a numerical value for the current stimulus (see Methods).

While artificial grammar-learning research generally indicates better learning for adjacent versus non-adjacent regularities, evidence suggests that non-adjacent regularities can be learned nonetheless (Endress & Bonatti, 2007; Gomez, 2002; Pacton et al., 2015; Wilson et al., 2020). However, adjacency effects may not necessarily manifest in our study due to key methodological differences from typical statistical/artificial grammar-learning studies, such as the presence of an explicit initial stimulus (S) and the absence of rapid stimulus sequences. In sum, our study design presents both potential advantages and challenges for regularity detection. For one, the active categorizations may facilitate regularity detection through more deliberate decision-making processes. Conversely, the repeated stimulus presentation with each categorization decision could increase its salience, potentially directing attention away from previous categorization outcomes and impeding regularity learning.

### Using temporal regularity in category learning: Type II structure

In visual category learning, Type I and Type II structures are typically learned more rapidly than Type VI structures, often interpreted as evidence for rule-based processing (e.g., Shepard et al., 1961; Nosofsky et al., 1994a; Lewandowsky, 2011; Mathy & Bradmetz, 2011; Love, 2002; Kruschke, 1992; Wills et al., 2015; Wills et al., 2020; Lamberts, 1995; Anderson, 1991; Nosofsky, 1991a; Nosofsky, 1991b; Schlegelmilch et al., 2022; Bruner et al., 1956; Martin & Caramazza, 1980). While learning a Type I regularity structure seems plausible, this may not hold true for more complex temporal rule-based structures if temporal regularities are learned incidentally. As illustrated in Fig. 1, a solution in the Type II (XOR) structure is based on two dimensions (e.g., size and color). For example, black-small and white-large objects belong to category A, whereas small-white and black-large objects belong to category B. A recent successful model of explaining learning trajectories in Type II (see Schlegelmilch et al., 2022) assumes that participants combine two partial Type I rules, namely, if the stimulus is black, then a small object is assigned to category A and a large object to category B (rule 1 for black stimuli), but if the stimulus is white this rule needs to be reversed (rule 2 for white stimuli). However, this may be more difficult when the Type II pattern involves three separate tasks, because participants have to remember information from earlier tasks to predict the outcome of the third one. This would be easier if all the necessary information was shown on the screen at the same time.

In particular, a temporal Type II structure requires three consecutive categorizations for the same stimulus with outcomes O$^1$: A versus B, O$^2$: C versus D, and O$^3$: E versus F. Here, the first two categories (O$^1$ & O$^2$) disjunctively predict the third one (O$^3$), such that categorization outcomes AC$\rightarrow$E and BD$\rightarrow$E but AD$\rightarrow$F and BC$\rightarrow$F, also illustrated in Fig. 2. Note, procedurally, this replaces the unrelated task X shown in Fig. 3, with a third categorization task O$^3$. Thus, the design adds further complexity to the Type I structure discussed above, as there are now three instead of two interrelated decision outcomes, and O$^2$ predicts O$^3$ contingent on O$^1$. While some evidence suggests learning temporal Type II structures in serial reaction time paradigms to some extent (e.g., Lazartigues et al., 2022), our task’s specific requirements may present unique challenges. Notably, maintaining not just one but two previous outcomes in working memory poses a cognitive demand on predicting the third outcome based on a Type II rule. As noted, thus, learning a temporal Type II structure might also be more challenging than in the visual domain, where all features appear simultaneously, potentially facilitating the discovery of such regularities.

## Study 1

Study 1 examined participants’ ability to detect and utilize a temporal Type I rule, mirroring observations in visual category learning. The experimental paradigm is divided into three phases, (1) a learning phase, where participants viewed idiosyncratic stimuli each followed by multiple decision tasks (e.g., S$\rightarrow$O$^1$[A vs. B]$\rightarrow$O$^2$[C vs. D]$\rightarrow$X) with immediate feedback. (2) Phase two is a test phase where we present novel visual stimuli to assess whether the participants use the outcome of task O$^1$ to predict the outcome of task O$^2$ (i.e., generalization the temporal Type I regularity). (3) The third phase is a recall phase where the participants only perform task O$^2$ (i.e., S$\rightarrow$O$^2$) without feedback to evaluate memory for stimulus-category associations. We hypothesized, as pre-registered on OSF (https://osf.io/qdngu), that the adjacent regularity condition (S$\rightarrow$O$^1\rightarrow$O$^2\rightarrow$X) would yield higher accuracy for O$^2$ categorizations compared to the control condition during the learning phase. For the test phase, we predicted the generalization of the Type I regularity to novel targets, with consistent decisions (C after A, D after B) occurring more frequently than chance in the regularity condition. A non-adjacent condition (i.e., S$\rightarrow$O$^1\rightarrow$X$\rightarrow$O$^2$) was later added for comparison. As this condition was not pre-registered, the data collection was done separately. This design allowed us to investigate differences in accuracy between Type I and unstructured temporal regularity, assess generalization to novel targets, and explore the impact of adjacency on learning temporal regularities. The analyses include an exploration of a subsequent outcome recall phase without feedback, where participants were presented with stimuli and asked to recall only the second outcome of the sequence (i.e., S$\rightarrow$O$^2$). This phase allows us to assess whether participants’ ability to memorize stimulus-category associations differed between conditions with and without temporal regularity.

### Method

#### Participants & sample size

We recruited 74 German-speaking students of the University of Bremen. The participants were randomly assigned to the control and adjacent conditions. To determine the sample size, according to the pre-registration, we implemented the Sequential Bayes factor method with maximal *N* design (Schönbrodt & Wagenmakers, 2018). We determined a minimum $(N_{min}) = 30$ participants per condition and a maximum of $(N_{max}) = 150$ participants in total. Once the minimum was achieved, we calculated the Bayes factors (BF) for our hypotheses tests on the test phase (new stimulus) and stopped when reaching above 10 or below 0.1 (Wagenmakers, 2007), after step-wise increments in sample size by 10 (5 in each condition). The BF’s and $N_{max}$ were based on a one-sample *t*-test (see test phase analyses), using G*Power (Faul et al., 2007) to determine the latter (Power >90% assuming $\upalpha$ = 5% and *d* = .34). After finding initial support to our hypothesis we recruited 37 participants for the non-adjacent condition (in total: female = 76, male = 31, non-binary = 1; *M*(age) = 25, *SD* = 5). The procedure, on average, took *M* = 47.98 minutes, *SD* = 13.12, and the participants received course credit or 10 euros as compensation. Of the 111 participants, three indicated that their data should not be used for analysis (pre-registered). In total, 108 participants remained, with N(control)= 35, N(adjacent)=36, and in N(non-adjacent)= 37.

**Table 1:** Study 1 Learning targets

S	O$^1$	O$^2$	X
	Herf	Krill	12
	Herf	Krill	25
	Herf	Krill	7
	Herf	Krill	33
	Jonth	Wask	6
	Jonth	Wask	17
	Jonth	Wask	43
	Jonth	Wask	36

*Note.* The table shows targets for the adjacent regularity condition, S$\rightarrow$O$^1\rightarrow$O$^2\rightarrow$X. S = Stimulus, O$^1$ = categories decision one, O$^2$ = categories decision two, X = criterion values of irrelevant task. In the non-adjacent condition, the decision sequence changed to S$\rightarrow$O$^1\rightarrow$X$\rightarrow$O$^2$ (cet. par). In the control condition, Herf and Krill were paired in 50% of the cases (same for Jonth and Wask), with S$\rightarrow$O$^1\rightarrow$O$^2\rightarrow$X

#### Materials & procedure

After providing informed consent, participants received basic instructions and training examples, followed by three control questions to ensure task comprehension. Incorrect answers prompted a summary of the training phase instructions. The experiment consisted of a training phase, a generalization test phase, and a final recall test. Upon completion, participants rated task difficulty and their diligence, could describe any strategies used, and were given the option to withdraw consent. All studies were approved by the Ethics Committee of the University of Bremen.. The experiment, created using jsPsych (De Leeuw, 2015), was conducted in single-seat cubicles. Participants were tasked with analyzing “elements” on a distant planet, predicting three outcomes for each element detected. All materials are available on the OSF repository: https://osf.io/ymu4b/?view_only=b05942441669406eb04673453094a9ee

During training, eight stimuli (Table 1; designed by Freepik or taken from Flaticon.com) were presented in separate trials. As illustrated in Fig. 3, in each trial, a stimulus was presented and participants performed a sequence of decisions for this stimulus. Each decision sequence comprised three predictions (two categorization tasks and one estimation task) on separate slides, with the visual stimulus (S) always present. As labels for the binary categorization outcomes, we chose fictitious labels (e.g., task 1: Herf vs. Jonth, task 2: Krill vs. Wask). The first decision (e.g., Herf vs. Jonth) was prompted by “Predict the outcome of Analysis 1” below the stimulus. Participants performed the categorization task by clicking on one of the two presented category labels and received immediate feedback (e.g., “Correct! The prediction is Herf”), after which the prompt for the second task followed (e.g., “Predict the outcome of Analysis 2”), followed by the third task. Which category a stimulus was assigned to was randomly counterbalanced between participants for each stimulus. In addition, the presentation order of the category buttons on the screen was randomly flipped between stimuli to prevent motor learning. For each participant, the eight stimuli were randomly selected from a set of twelve, with the remaining four used in the test phase as novel stimuli. During training, the eight stimuli were presented in a random order in ten blocks (80 trials).

In the adjacent condition, the second decision was another categorization task which followed the same procedure as the first categorization task. The third task was an irrelevant task. In this task, participants had to estimate a value, ranging between 1 and 50, which was labeled the KIV value (a fictitious label). The participants entered their estimate in a text box and pressed the space bar. Afterwards, they received feedback on whether their estimation had been correct. The irrelevant task was added in anticipation of further conditions to keep the overall effort in learning the three-task sequence comparable. That is, in the non-adjacent condition, the irrelevant task was presented between the two categorization tasks. We chose an estimation task for this because we wanted to test the effects of adjacency (i.e., temporal distance) without introducing interference due to similar tasks. We address the potential implications in the Discussion.

The generalization test phase used the same procedure as the training phase, but presented four randomly selected stimuli from the training set(two from each O$^1$ category) and four novel targets (two assigned to each O$^1$ category), all presented in ten randomized blocks. However, feedback was provided for all stimuli regarding O$^1$ categories but not for O$^2$ categories to test whether the choices regarding O$^2$ for novel items are consistent with the learned temporal regularity predicted by the just-observed O$^1$ outcome. This also rules out that behavior is affected by forgetting about the S$\rightarrow$O$^1$ associations for old targets.

A recall phase followed, presenting all eight previously trained objects in random order (four repeated blocks, no feedback), however, asking the participants to only perform the second categorization task (O$^2$). We neither included novel stimuli nor information about the first task (O$^1$). We did this because we wanted to test whether learning of an O$^1$ $\rightarrow$O$^2$ regularity affects the accessibility of the stimulus outcome association for task 2 (S$\rightarrow$O$^2$) when only providing the stimulus, relative to the control condition. For example, noticing the regularity between the outcomes of task 1 and task 2 could lead to a strategic neglect of encoding the association between the visual stimulus and the outcome of task 2. Thus, such a strategy would still allow informed/generalized responding to novel stimuli when information about O$^1$ is present (generalization test phase), but might also reduce performance in the recall phase, because overt information regarding O$^1$ is absent, and participants need to use the visual stimulus for making the categorization. We explore performance in the recall phase in terms of accuracy and response times in the analyses further below.

#### Data cleansing

We aimed to analyze behavior when learning occurred, initially planning to exclude participants with near-chance performance in the first categorization (O$^1$) at the end of training. Our pre-registered method would have excluded participants not significantly deviating from 50% accuracy in the last 24 trials of task 1. However, this criterion proved too strict, potentially excluding 41 participants.

To address this, we employed a Bayesian latent class model to classify participants into guessing, medium, and high-accuracy groups based on the final three (exclusion method 1, EM1) or two (EM2) training blocks. We set the priors of each group mean probability as, respectively, $\upphi _{guessing}=.5$, and $\upphi _{medium}$ and $\upphi _{high}$, with the latter two drawn from a uniform distribution between .5 and 1. The models were assigned to participants using a trans-dimensional MCMC method with uniform Dirichlet prior, passed to the individual level via categorical samples (for similar applications, see Schlegelmilch & von Helversen, 2020; Zeigenfuse & Lee, 2010). We calculated how often each participant was assigned to each model in all iterations, serving as a measure of confidence (e.g., 80% if assigned to the guessing model in 80% of all iterations).

We then conducted analyses without exclusions and with the two exclusion methods based on the accuracy of the last three (EM1) and last two (EM2) blocks, both using an 80% confidence criterion for assigning participants to the guessing group, respectively, excluding 25 and 21 participants. These methods did not affect the main conclusions, but only minor secondary results. We, therefore, report the results using the full sample size and highlight the exception in the corresponding analysis.

### Result

#### Training accuracy

**Fig. 4:**
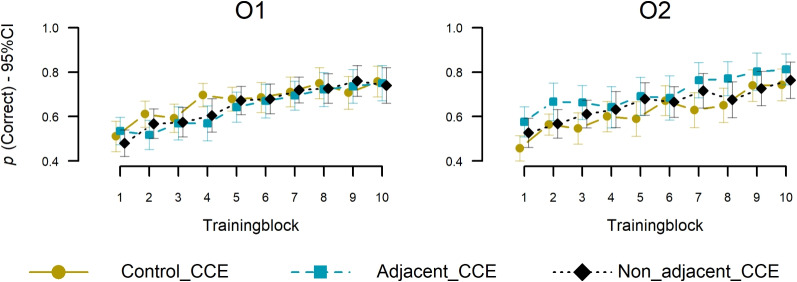
Mean accuracy during the training phase. Accuracy (y-axes) over training blocks (x-axes) with 8 stimuli per block. The left panel shows accuracy in the first task and the right panel in the second task. Error bars indicate 95% CIs of individual means

Regarding the training phase, we hypothesized that an existing adjacent regularity between O$^1$ and O$^2$ increases the learning speed of O$^2$, compared to the control condition (Type I adjacent vs. control), but that learning of O$^1$ should be equal between both. Similarly, we hypothesized that an adjacent regularity between O$^1$ and O$^2$ leads to quicker learning of O$^2$ compared to the non-adjacent condition without affecting O$^1$ accuracy (Type I adjacent vs. Type I non-adjacent). We test both hypotheses separately below, as we assessed the latter Type I non-adjacent condition to supplement the Type I adjacent versus control investigation. Figure 4 shows the corresponding learning curves in the three conditions, calculated as objective accuracy for the participant’s predictions of O$^1$ (left panel) and O$^2$ (right panel), averaged within the ten repeated training blocks. As can be seen, while O$^1$ accuracy appeared to be very similar between the three conditions, O$^2$ accuracy seemed higher in the adjacent condition, compared to both the non-adjacent and control conditions.

To test for corresponding effects regarding the Type I adjacent and the control condition, we performed a mixed-effects logistic regression on the binary responses (1=correct), entering condition (adjacent vs. control), decision task (O$^1$ vs. O$^2$), and training blocks (continuous, mean-centered) and all their interactions as fixed effects (using the R package afex; Singmann et al., 2023) with Type III LRT tests (full vs. reduced model). For consistency and comparability, we always implemented the same random effects structure for the training data that converged in all analyses or did not otherwise lead to model identification issues (e.g., under different exclusion criteria). Therefore, we assume by-participant and by-stimulus random intercepts and by-participant random slopes for the main effects of blocks and tasks. First, we found a significant main effect for blocks, $\chi {2}$ (1, 70) = 56.05, *p* < .001, reflecting the increase in accuracy over time. Importantly, as hypothesized, there was a significant interaction between condition and decision task, $\chi ^{2}$(1, 70) = 14.25, *p* < .001, indicating that task-specific accuracy differed between the adjacent and control conditions. There was no main effect for condition, $\chi ^{2}$(1, 70) = 2.19, *p* = .139, or decision task, $\chi ^{2}$(1, 70) = 2.43, *p* = .119, and no interactions between decision task and blocks, $\chi ^{2}$(1, 70) =1.07, *p* = .30, condition and blocks, $\chi ^{2}$(1, 70) = 1.48, *p* $=$ .223, or a 3-way interaction, $\chi ^{2}$(1, 70) = 0, *p* = .983.

In post-hoc analyses on the mixed-model estimates (log-scale; R package: Emmeans; Lenth et al., 2019), we further confirmed that the interaction between decision task and regularity condition, as hypothesized, was driven by higher accuracy in the adjacent condition compared to the control condition on O$^2$, $z = 2.60$, $p =.009$, *Mdiff* = 0.65, *CI95* = [0.16,1.14], while there was no difference on O$^1$ accuracy, *z* = -0.34, *p* = .73, *Mdiff* = -0.06, *CI95* = [-0.41, 0.29]. Furthermore, as expected, accuracy was higher on O$^2$ compared to O$^1$ within the adjacent condition, *z* = -3.83, *p* < .0001, *Mdiff* = -0.5, *CI95* = [-0.75,-0.24]. In contrast, there was no difference on O$^2$ than on O$^1$ in the control condition, *z* = 1.69, *p* $=$ .09, *Mdiff* = 0.21, *CI95* = [-0.035,0.46].^1^

Next, we tested our hypotheses regarding the role of adjacency, conducting the same analysis as above but comparing adjacent and non-adjacent Type I conditions. We found significant main effects of blocks, $X^{2}$ (1, 72) = 56.41, *p* < .001, as well as decision task, $X^{2}$ (1, 72) = 10.15, *p* < .001, such that O$^2$ accuracy was generally higher than O$^1$. Importantly, as hypothesized, there also was an interaction between condition and decision task, $X^{2}$(1, 72) = 6.92, *p* $=$ .009, indicating that the relative accuracy of O$^1$ and O$^2$ depended on the condition. There was no significant main effect of condition, $X^{2}$(1, 72) = 0.75 *p* = .386, and no interactions between decision task and block, $X^{2}$(1, 72) =0.18, *p* = .670, condition and block, $X^{2}$(1, 72) = 0.29, *p* = .593, or a three-way interaction, $X^{2}$(1, 72) = 1.41, *p* = .236.

The corresponding post-hoc analyses on the central interaction between the condition and decision task revealed that, unlike in the above-reported adjacent condition, there was no difference between O$^1$ and O$^2$ in the non-adjacent condition *z* = -0.43, $M_{diff}= -0.05$, $p =.67$, CI95= [-0.28,0.18]. This difference seemed unlikely due to the fact that O$^2$ was presented at different positions in the two conditions (i.e., the second task in adjacent and the third task in non-adjacent), because when comparing the performance of the irrelevant task (e.g., the third task in adjacent and control, and second task in non-adjacent), the result instead suggested no difference (see further Supplements: S2). While the above within-condition effects were conclusive, however, the corresponding between-condition comparison was not when comparing performance in task 1 and task 2 separately. Regarding O$^1$, there was no significant difference between adjacent and non-adjacent conditions, *z* = -0.167, $M_{diff}= -0.03$, $p =.87$, CI95= [-0.42,-0.35]), and also not regarding O$^2$,*z* = 1.55, $M_{diff}= 0.41$, $p =.12$, CI95= [-0.11,0.93], which may be due to statistical power, as we aimed to test the effects in the final test phase.^2^

Overall, thus, the result of improved O$^2$ learning speaks for the hypothesis that participants learned the adjacent category-category regularities beyond stimulus-category associations (compared to control). Moreover, O$^2$ performance seemed slightly worse relative to O$^1$ in the non-adjacent condition compared to the adjacent condition, providing some evidence for a somewhat weaker effect of inter-category regularities in the former, which we further discuss in light of the generalization results below. Note, we conducted a similar analysis for the old targets in the test phase. Similarly to the training phase, we found an increase in performance in the predicted task, O$^2$, in the adjacent condition compared to the control condition, and there was no difference in performance between the adjacent and non-adjacent conditions. The details and results of the analysis are reported in the supplementary materials S1.

#### Test phase transfer: novel targets

**Fig. 5:**
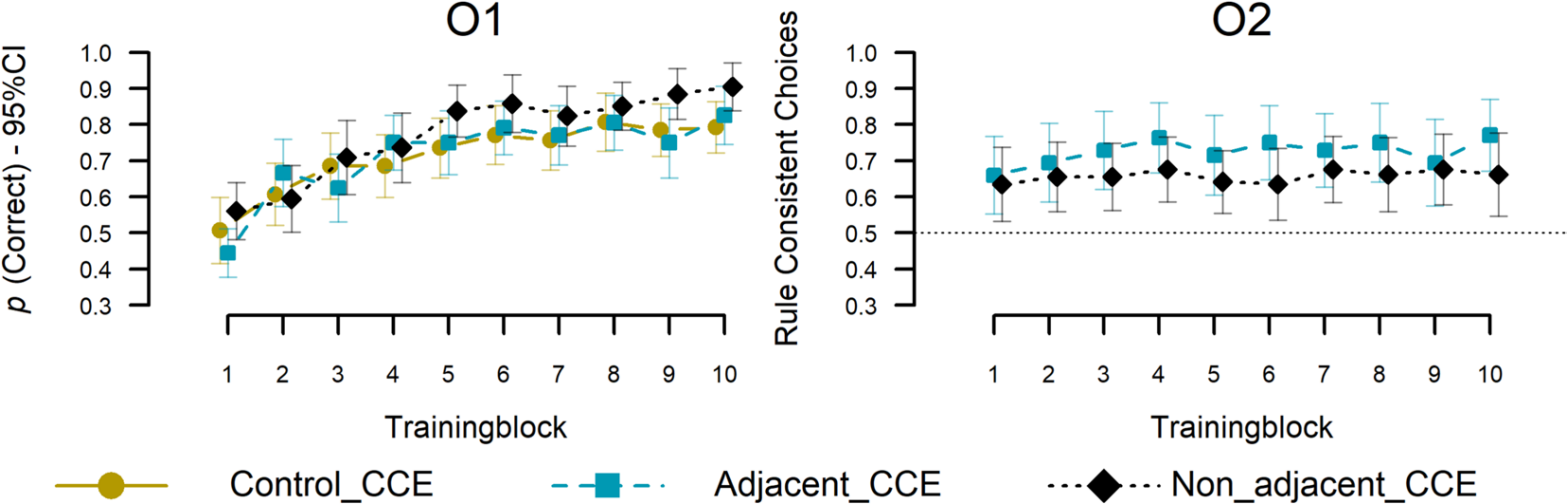
Test phase (new objects). The left panel shows accuracy (y-axis) over test blocks (x-axes) with 8 trials per block for categorizations in task 1. The right panel shows the percentage of rule-based categorizations (y-axis) over test blocks (x-axis) with 8 trials per block for categorizations in task 2 against the chance level (50% accuracy). Error bars indicate 95% CIs of individual means

Regarding the novel targets, we hypothesized that Type I consistent predictions would be more frequent than chance (50%) in the adjacent and non-adjacent conditions but more frequent in the former than in the latter. We define “Type I-consistent” as choices that adhere to the same category-category regularity as observed before; that is when feedback O$^1$ was “A”, then an O$^2$ response of “B” would be consistent (e.g., Krill always followed Herf), and equally when O$^1$ was “B,” and O$^2$ was “D”. Figure 5 (right) depicts the corresponding average choice consistency over the test blocks for the adjacent and non-adjacent conditions. Note, since there was no regularity in the control condition, we did not include this condition in the comparison but tested our first hypothesis using a Bayesian one-sample t-test (R package BayesFactor, default priors; Morey & Rouder, 2023) comparing the participants’ percent consistent choices against 50% (guessing) in each condition, and a two-sample t-test for comparing both regularity conditions. As predicted, we found conclusive evidence of regularity consistent choices in the adjacent condition, $BF_{10} = 1028.63$; *M* = 72.57, *SD*= 44.63), indicating that participants successfully generalized the regularity to novel targets. Similarly, we found conclusive evidence for the same trend in the non-adjacent condition, $BF_{10} = 106.39$; *M* = 65.74, *SD*= 47.47, indicating that participants successfully abstracted and generalized the regularity to novel targets in both conditions. Interestingly, there was no evidence for a difference in regularity-consistent responses between adjacent and non-adjacent conditions, $BF_{10} = 0.42$, remaining inconclusive. At a minimum, the results suggest, however, that inter-category regularity was acquired in both conditions.

#### Explorative analyses: recall phase

The recall phase aimed to assess whether the introduction of the Type I regularity in the decision sequence affected the overall recall of the S$\rightarrow$O2 association. If participants learned a Type I regularity, they might have less strongly memorized this association, as it became redundant with the O$^1\rightarrow$O$^2$ regularity in predicting the second outcome. During the recall phase, this could manifest as lower accuracy in the regularity conditions compared to control, or longer response times due to participants recalling O$^1$ to predict O$^2$ indirectly.

In brief, we observed little to no differences in the mean accuracy in task O$^2$ in the control ($M = 68.3, SD = 17.7$), the adjacent ($M = 74, SD = 21.5$) and the non-adjacent condition ($M = 73.3, SD = 22.6$). We still performed a mixed-effects logistic regression model with conditions and blocks as fixed effects with by-participant and by-stimulus random intercept. We found no significant main effect of condition, $X^{2}$(1, 106) =2.68, *p* = .262 or blocks,$X^{2}$(1, 107) =1.09, *p* = .298) and no interaction effect between blocks and condition, $X^{2}$(1, 106) =1.05, *p* $=0.59$. The above results were also partially supported in regards to the reaction times (in seconds) in control ($M = 2.21, SD =.97$), the adjacent ($M = 1.98, SD =.76$), and the non-adjacent condition ($M = 1.73, SD =.78$). We found inconclusive results comparing reaction times using a Bayesian t-test between the adjacent and control condition, $BF_{10} = 0.42$, and inconclusive evidence for the null hypothesis for the comparison between the adjacent and non-adjacent condition, $BF {10} =0.55$. Thus, the evidence suggests that a Type 1 category-category adjacent regularity does not necessarily impair learning the stimulus-category associations (S$\rightarrow$O$^2$) compared to the control condition.^3^

#### Discussion Study 1

The results of Study 1 generally supported the hypothesis that participants can learn Type I regularities between sequential decision outcomes, demonstrating a learning benefit for the second decision compared to a control condition without regularity. This conceptually parallels the observed ordinal difficulty between Type I (rule-based) and Type VI (unstructured) problems in visual category learning (Shepard et al., 1961; Nosofsky et al., 1994a; Lewandowsky, 2011). Accordingly, participants successfully generalized the Type I regularity to novel targets in a transfer phase. Inspired by artificial grammar-learning research (e.g., Wilson et al., 2020), we also examined whether temporal spacing between associated outcomes (adjacent vs. non-adjacent) affected learning and generalization of the Type I regularity. Although slight trends suggested quicker learning and more regularity-consistent generalization with adjacent regularities compared to non-adjacent ones, the results were generally inconclusive. Due to the relatively small effects between the latter conditions, this result warrants further research with larger sample sizes to draw more definitive conclusions.

The current results demonstrate that effects observed in Type I versus Type VI visual category structures generalize to temporal regularities, extending theoretical perspectives on cognitive processes driving learning and outcome generalization. This finding bridges the gap between visual category learning and learning of temporal regularities, suggesting potentially shared underlying mechanisms. Importantly, the observed learning benefits did not impair memory for stimulus-category associations, as evidenced by the exploratory recall task. The results thereby highlight the current design’s potential to dissociate effects concerning rule learning and generalization from those concerning memorization, opening promising avenues for future research on the cognitive processes underlying temporal regularities and category learning.

Our study further reveals that participants readily learned Type I regularities in both adjacent and non-adjacent conditions, suggesting a robust ability to detect and utilize temporal patterns. The absence of evidence for differences between adjacent and non-adjacent conditions seems to underscore the participants’ capacity to compensate for potential difficulties induced by temporal distance. As Wilson et al. (2020) points out, there seem to be certain conditions identified in artificial grammar-learning, which moderate effects of adjacency by bringing non-adjacent elements attentionally or perceptually closer together (e.g., when the sequence begins and ends on the relevant elements, known as edge effects). In our design, this could further concern the choice of the irrelevant task, which was not a categorization but an estimation judgment. Thereby, participants could have more easily recognized the relation between the two categories than perhaps would be expected if the irrelevant task was a categorization task as well, warranting further research on the processes involving selective attention. At minimum, the result implies that participants could retain a corresponding representation of previous outcomes in working memory despite intervening irrelevant task. This insight also concerns Study 2, in which we test whether a Type II regularity can also be discovered in a sequence of decisions, as suggested by research on visual category learning (e.g., Shepard et al., 1961; Nosofsky et al., 1994a; Lewandowsky, 2011). That is, in this problem, finding a rule solution would require integrating category outcomes from two previous decisions to predict a third categorical outcome.

## Study 2

### Method

Study 2, pre-registered on OSF (https://osf.io/xp48s), employs similar methods to Study 1, with the key difference being the implementation of a Type II structure involving three binary categorical tasks O$^1$ (categories A and B), O$^2$ (C and D), and O$^3$ (E and F) instead of two. To maintain a consistent sequence length with Study 1, we replaced the irrelevant task by the relevant categorization. Figure 2 illustrates the categorical associations between the outcomes, demonstrating how O$^1$ and O$^2$ disjunctively predict O$^3$ in an XOR structure. The critical test of our hypothesis regarding a learning benefit for Type II focuses on O$^3$, which is compared to an unstructured control condition as in Study 1. We hypothesized that generalization of the regularity to novel targets would lead to above-chance, rule-consistent choices in a subsequent test phase. Overall, the study implements a 2 (Regularity: Type II vs. Control; between-subjects) x 3 (Decision Task: task 1 vs. 2 vs. 3; within-subjects) mixed factorial design.^4^

#### Participants

We recruited 70 English-speaking participants on Prolific Academic, with 2 participants choosing to withdraw consent (Male = 40, Female = 28; *M*(age) = 32, *SD* = 7), randomly assigned to the two conditions, with N(control)= 33, and N(type II)=35. They received £4.00 as basic compensation and an additional bonus, contingent on the number of correct choices during the training phase (£2 max), with the average bonus being £1.68. The study duration was *M* = 31.32 minutes, *SD* = 16.28 minutes.

The sample-size rationale followed the Sequential Bayes factor method with maximal *N* design (Schönbrodt & Wagenmakers, 2018) based on the hypothesis test in the test phase for novel targets, we determined a minimum $(N_{min}) = 30$ participants. We continued the sampling until a BF>10 or BF<0.1. For determining $N_{max}$, we simulated the category decisions in the Type II condition according to a small effect p =.6 rule-consistent responding in the Type II condition (i.e., the mean probability of the consistent response based on a Binomial distribution for each individual in 16 trials [4 novel targets * 4 blocks]), then conducted a one-sample Bayesian t-test on the simulated data against mean = .5. We repeated the procedure 500 times, with different sample sizes, and obtained a BF>10 in 90.4%, with 60 participants in each condition, which we set as $N_{max}$. Post-registration, we noticed that simulations assuming no effect rarely reached BF<0.1 for the given number of stimuli and blocks, regardless of participant numbers. We adjusted the stopping criterion to BF<1/3 for the generalization hypothesis test. After increasing the sample to 70 participants, we found BF < 1/3 and stopped sampling.

#### Materials & procedure

The experiment was created using jsPsych (De Leeuw, 2015) and conducted online. The procedure and trial-wise decisions were identical to that of Study 1, except for changing the problem structure, replacing the irrelevant task with the additional categorization, and not testing a final outcome recall. The implementation of the critical Type II structure in the three decision tasks is illustrated in Table 2. In each, participants classified the object into one of two categories ( O$^1$: Herf vs. Jonth; O$^2$: Krill vs. Wask; O$^3$: Thesh vs. Aurek). In the control condition, there was no regularity between the outcomes of the three tasks.

**Table 2:** Task Design - Type II Regularity

Object	Task 1	Task 2	Task 3
	Herf	Krill	Aurek
	Herf	Krill	Aurek
	Herf	Wask	Thesh
	Herf	Wask	Thesh
	Jonth	Wask	Aurek
	Jonth	Wask	Aurek
	Jonth	Krill	Thesh
	Jonth	Krill	Thesh

Task 1-3 refer to subsequent decisions for the same object. “Herf” and “Jonth” refer to category outcomes in task 1, “Krill” and “Wask” to outcomes in task 2, and “Thesh” and “Aurek” to outcomes in task 3, with Type II structure to predict task 3

Participants completed two phases: training and test. The training phase involved twelve blocks of eight objects, following the same categorization decision method as Study 1. We extended the training phase by two blocks to provide sufficient learning opportunity. The test phase presented four old and four novel targets, randomized within four repeated blocks. Old targets were randomly selected from the training phase, representing each unique combination of the critical Type II O$^1$ and O$^2$ category combinations (e.g., Herf-Krill, Jonth-Wask, both leading to Aurek). The four novel targets replicated these combinations to assess decision consistency with the Type II structure. Participants received feedback for tasks 1 and 2, but not for task 3, mirroring the approach in Study 1. To motivate learning, participants could earn a bonus payment based on performance in each phase. In the training phase, correct responses earned points (maximum 2880), with 5 pence awarded per 288 points, potentially yielding an additional 50 pence. Bonus thresholds of 1440 and 2160 points triggered 25 and 50 pence payments, respectively. In the test phase, participants received 50 pence for reaching 400 points (from a maximum of 800) and £1 for exceeding 600 points.

#### Data cleansing

In Study 2, we followed the same procedure as Study 1 and generally reported the results without exclusions, as applying the previously described exclusion criteria did not affect the main conclusions. The total number of participants excluded based on the EM1 (three training blocks and 80% confidence) and EM2 (two training blocks and 80% confidence) methods were 32 and 0, respectively. Since the EM2 method did not exclude any participants, we highlight the differences between the results based on the filtered data using the EM1 exclusion method.

### Results

#### Training accuracy

**Fig. 6:**
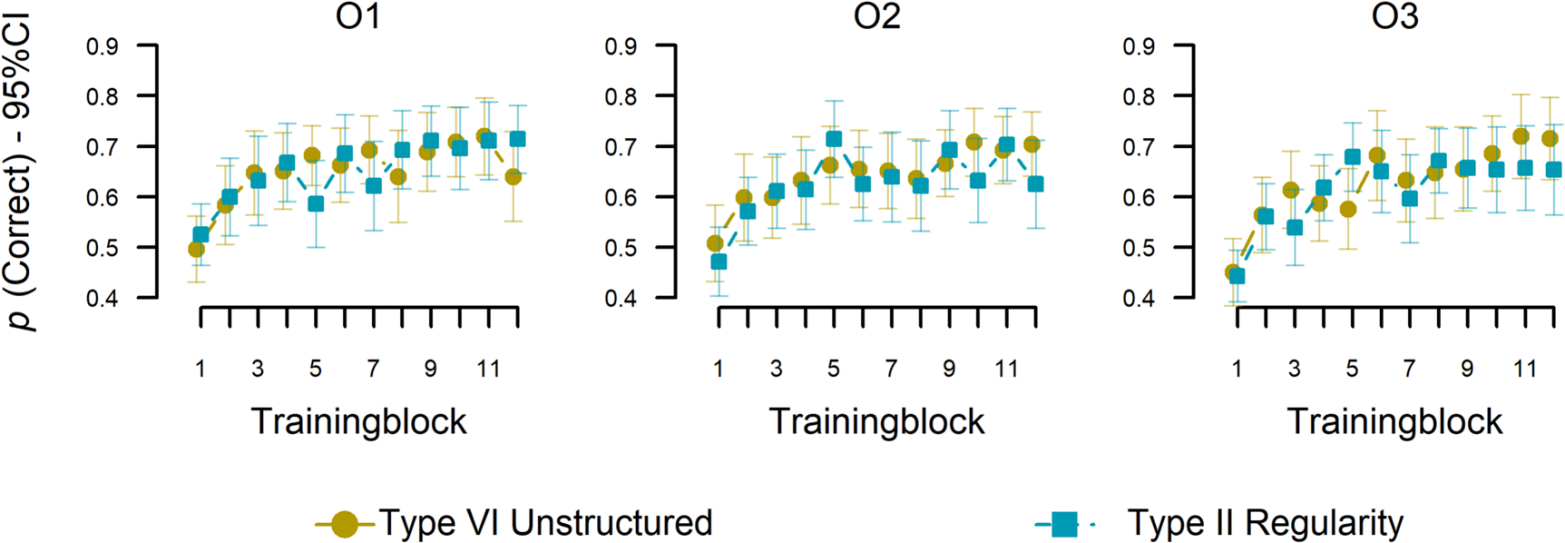
Training phase mean accuracy. Accuracy (y-axes) over training blocks (x-axes) with 8 trials per block) for O$^1$ categorizations (left), O$^2$ (middle), and O$^3$ (left). Error bars indicate 95% CIs of individual means

Our main goal was to test whether participants could learn the classical Type II structure when embedded in a sequence of categorization tasks. We hypothesized that learning would be better in Type II compared to a decision sequence without structure (control). Figure 6 shows the basic results, suggesting no difference in learning accuracy on O$^3$ between Type II and control. We performed a mixed-effects logistic regression (R package afex; Singmann et al., 2023) on accuracy using Type III LRT tests with training blocks, condition, and decision task (O$^1$ vs. O$^2$ vs. O$^3$) as fixed effects, including by-stimulus and by-participant random intercepts and by-participant random slopes for the main effects of blocks and task. As also suggested in Fig. 6, we found significant main effects of decision task, $\chi ^{2}$(1, 66) = 9.94, *p* = .007, and blocks, $\chi ^{2}$ (1, 67) = 22.67, *p* < .001, showing that accuracy increased over the training trials. There was no significant effect for the main effect condition $X^{2}$ (1, 67) = 0.02, *p* $=$ .892. Importantly, there was no significant interaction between condition and decision task, $X^{2}$(1, 66) = 0.98, *p* = .61, indicating that O$^3$ accuracy, indeed, did not differ between the Type II and the control condition. There were also no interactions between decision task and blocks, $X^{2}$(1, 66) = 2.55, *p* = .279, condition and blocks, $X^{2}$(1, 67) = 0.09, *p* = .769, or a 3-way interaction, $X^{2}$(1, 66) = 2.82, *p* = .244.^5^

The training results suggest that learning Type II versus control does not reproduce the ordinal difficulty trends observed in visual category learning. In other words, the result show that there is some learning, but they do not support the hypothesis that participants abstracted an XOR rule in the sequence of categorization decisions, which would predict a performance benefit over the Type VI unstructured condition.^6^ The old targets’ performance in the test phase shows the same patterns as the training phase (see supplementary materials S3). However, similar to the results comparing adjacent versus non-adjacent conditions in Study 1, it remains possible that participants could still generalize the Type II regularity in the test phase.

#### Testing accuracy: novel targets

**Fig. 7:**
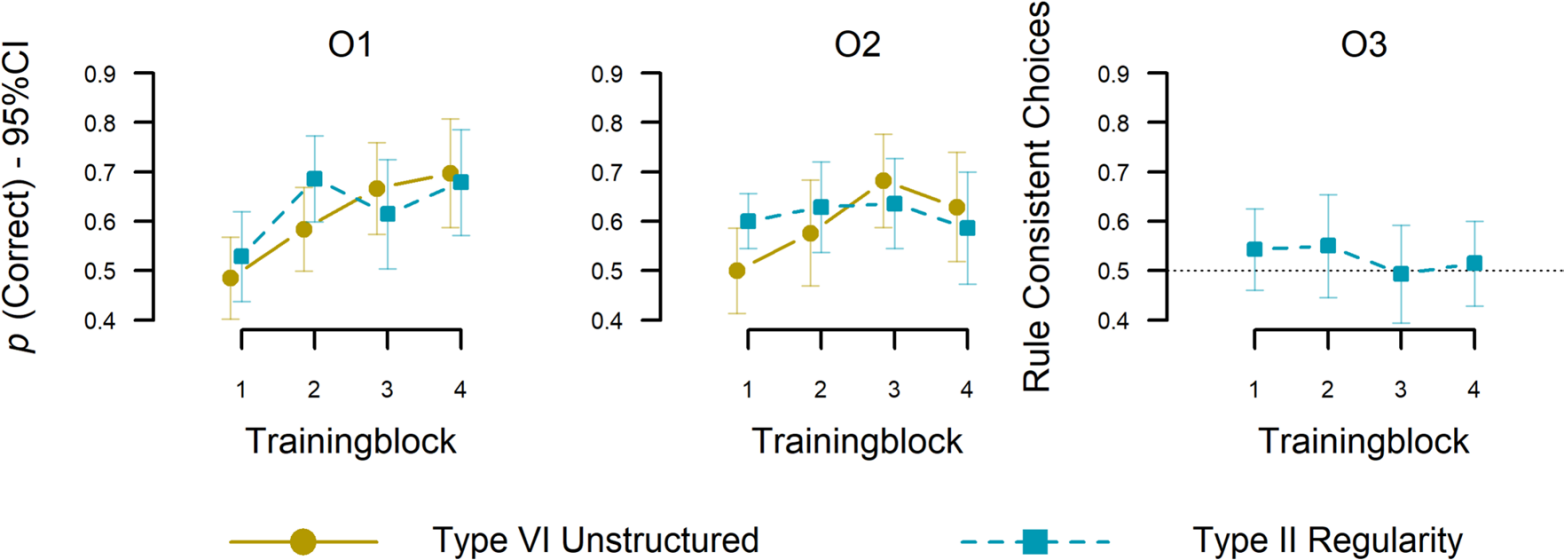
Test phase (new targets). Accuracy (y-axes) over test blocks (x-axes; 4 trials each block) for O$^1$ categorizations (left) and O$^2$ (middle). Rule-based categorization Percentage (y-axis) over test blocks (x-axis; 4 trials each block) for O$^3$ categorizations (right) against chance level (50% accuracy). Error bars indicate 95% CIs of individual means

Finally, we tested whether participants in the Type II condition, despite comparable training performance to the control, could generalize the regularity to novel objects. To assess this, we calculated decision consistency in O$^3$ responses according to the Type II structure—specifically, whether participants followed the same pattern of O$^3$ predictions based on O$^1$ and O$^2$ outcomes, which were provided via feedback. Generalization would be indicated by above-chance responding (greater than 50%) for the four novel targets. Figure 7 (right panel) shows the average choices across test blocks. We averaged responses for each participant and conducted a one-sample Bayesian t-test against 50% (guessing). The results provided conclusive evidence against our pre-registered hypothesis that participants applied (i.e., generalized) a temporal Type II regularity, because average rule consistency was indifferent from guessing, $BF_{10} = 0.24$. Thus, the transfer results reveal that participants failed to abstract the temporal regularity beyond the specific stimuli encountered during training.

#### Discussion Study 2

In Study 2, we tested whether participants could discover and generalize a Type II regularity embedded in a sequence of three consecutive categorization decisions. Compared to a control condition without regularity, the results support the Null hypothesis that learning performance in both conditions was equal and that participants did not generalize the Type II regularity to novel targets. Thus, conceptually, this result stands in contrast to the classical ordinal pattern observed in visual categorical learning, in which Type II is substantially easier to learn than Type VI (Shepard et al., 1961; Nosofsky et al., 1994a; Lewandowsky, 2011). In the following, we discuss the corresponding potential boundary conditions under which people can acquire abstract knowledge about categorization rules from different theoretical perspectives.

## General discussion

To summarize, we investigated whether humans can learn regularities between outcomes of sequential categorization decisions. We translated classical category learning designs, known as Type I (simple rule, Study 1) and Type II (disjunctive rule, Study 2; see Shepard et al., 1961; Nosofsky et al., 1994b) into temporal regularities (see also Lazartigues et al., 2022), such that one stimulus outcome could be predicted by sequentially preceding stimulus categories. We tested for learning benefits with temporal regularities compared to control conditions without regularities. Based on visual category learning insights, we generally expected that both temporal outcome regularities would result in better learning than the respective control condition. Study 1 also manipulated the temporal distance between associated task outcomes, and we expected stronger learning benefits for a Type I rule with adjacent than with non-adjacent relevant elements (see Lazartigues et al., 2022; Newport et al., 2004; Saffran et al., 1999; Deocampo et al., 2019; Wilson et al., 2020).

Study 1 revealed that a perfect Type I regularity between two categorical outcomes improved training performance on the predicted outcome compared to control and allowed participants to generalize the regularity to novel stimuli, robust in both adjacent and non-adjacent conditions. However, evidence for performance differences between adjacent and non-adjacent conditions in training and generalization was inconclusive. The evidence from Study 2 further suggests that the presence of a Type II (XOR) relation in the outcome sequence neither increased training performance nor led to regularity generalization compared to the Type VI baseline condition. In the following sections, we discuss theoretical implications, future directions, and limitations.

### Rule structure benefits in temporal category learning

The Type I benefit observed in the temporal Type I structure of Study 1 aligns with effects found in visual category learning. However, our findings in Study 2 diverge from the visual category learning literature, where Type II is typically easier to learn than unstructured categories (e.g., Shepard et al., 1961; Nosofsky et al., 1994a; Lewandowsky, 2011; Kurtz et al., 2013). This discrepancy in the temporal Type II problem raises critical questions about the boundary conditions and generality of existing learning theories. The well-replicated phenomenon of Type II superiority over Type VI in visual category learning makes our contrasting result particularly significant. It underscores the need to consider the modality of category predictors when determining whether people will extract rules or rely on memory-based learning, which adds to previous discussions on potential factors moderating type II learning (Kurtz et al., 2013; Schlegelmilch et al., 2022). Existing category-learning models, designed to predict both Type I and II advantages, are based on mechanisms forming visual predictor-outcome associations (for an overview, see Pothos & Wills, 2011). While it is technically possible to adapt these models by replacing stimulus features with previous category outcomes as predictors (i.e., everything seen so far can be used as predictor of the next category), such modifications would still predict a benefit for Type II over Type VI - a prediction not fully supported by our findings (i.e., only for Type I regularities). This highlights that additional assumptions are necessary that take into account learning processes specific to different modalities in category learning.

More generally, treating categories as “predictors” of other categories leads us to question what a “category” represents in the way people process our tasks. Traditionally, stimulus categories are described by “is-a” relations in concept formation (e.g., this object is a dog), while features are described by “has-a” relations (e.g., a dog has a tail; Goldstone, 1996). Similarly, past research distinguishes classification (using features to predict a category, such as size predicting an object’s category) from predictive inference (using a known category to predict features, which can influence behavior; see Markman & Ross, 2003; for similar trends in“cause” versus “effect” learning, see Buehner & Cheng, 2005; Lagnado et al., 2007).

While our tasks introduce categories in the usual way (“is-a” relations), we also find it useful to think of each item in the decision sequence in terms of its role—as either the “predictor” or the “to-be-predicted” element. For example, the category in task 1 is a target to be predicted at first, but later serves as a predictor of another category. This raises the question of whether the lack of Type II generalization in our study is related to whether the “to-be-predicted” elements are features or categories—possibly due to differences in how noticeable they are (Deng & Sloutsky, 2012; Johansen et al., 2015). This is an open question for future research.

One, perhaps more likely, reason for the presence of Type I but absence of a temporal Type II generalization might be the cognitive effort of keeping the necessary amount of information in working memory (one vs. two previous outcomes, respectively; for a related discussion, see Gureckis & Love, 2010). Thus, incorporating mechanisms of working memory into category learning models could, in principle, predict the null result regarding Type II, for example, when assuming that the focus of attention to process or maintain information is restricted to a single item (see Oberauer, 2019). Hence, it seems fruitful to test in future studies if visually presenting preceding outcomes, instead of hiding them after the respective decision, would allow participants to discover the rule that predicts the third outcome. Similar designs varying these factors, thus, might further allow a deeper investigation of the influence of cognitive effort for simultaneous maintenance and processing of information, and which could, conceptually, provide connections between the processes of category learning and performance in operation span working memory tasks (e.g., Conway et al., 2005).

Corresponding questions on whether people even consider the possibility of a temporal Type II regularity can also be found in a different strand of the categorization literature investigating how prior knowledge affects the learning of causal structures. That is, models that so far predicted both Type I and Type II benefits over Type VI problems build on the premise that participants deliberately consider complex category structures, and the models address the question how quickly they are learned, depending on the strength of learning rates and additional mechanisms that can lead to more effective learning in structured problems. Beyond strength, however, an alternative view is that participants do not consider every possible causal structure in the given problem situation. This has led to a *strength vs. structure* debate, as discussed by Lagnado et al. (2007). While the focus of our studies relied on the question whether people can from-scratch learn rule-based structures in categorization sequences via *incidental* learning, it seems reasonable to speculate that telling participants in advance about the possibility of complex temporal structures might improve their performance. While the role of prior knowledge in category learning seems understudied (see Bittermann et al., 2023), some previous research has shown in more natural prediction tasks that existing prior knowledge about possible causal, that is sequential, relations do affect learning and prediction behavior (see Waldmann & Holyoak, 1992; Kim & Rehder, 2011; Sloman & Lagnado, 2015). Thus, while our results present one condition in which Type II generalization was conclusively absent, our results do not rule out that participants *could* abstract a Type II structure under different conditions. Accordingly, participants may be able to detect a complex temporal regularity, such as an XOR structure, when they know what to look for. This would align with previous research (see Love, 2002) showing that participants learn category structures more effectively with intentional learning instructions than in incidental learning. In this vein, we believe that further extending the current design by conditions with more or less prior knowledge, varying instructions, and simultaneous presentation of predictive sequential information, as compared to different cognitive demands, seems to be a fruitful avenue to disentangle this perspective from explanations based on limited working-memory capacity and to hold the obtained results against benchmark results from visual category learning.

Furthermore, our findings relate to insights from statistical learning, artificial grammar-learning, and the serial reaction time paradigm, in which regularity benefits seem much stronger in Type I than in Type II (Lazartigues et al., 2022), there termed first-order and second-order transition probabilities, respectively. Although we did not directly compare both structures, warranting further investigation, their respective comparisons to control conditions suggest a parallel pattern in temporal categorizations. However, while we found no evidence for learning of the temporal XOR structure, studies on temporal regularity observed at least slight influences in an XOR structure, although only after extensive learning (Gureckis & Love, 2010) and only during test (i.e., not during acquisition; Lazartigues et al., 2022). Again, one reason for the diverging results could be that learning the temporal XOR structure in our task puts a higher load on working memory than learning an XOR structure in a serial reaction time task, as the latter usually involves observing a sequence of elements in quick succession. In contrast, our task required active categorizations of a visual stimulus as well as the processing of feedback. The longer spacing between the relevant elements may have increased demands on working memory (see also Gureckis & Love, 2010) and made attending jointly to the relevant elements more difficult, thereby impairing learning of the temporal structure.

### Adjacency in Type I learning and generalization

Inspired by research on artificial grammar-learning, which suggests better learning of adjacent than non-adjacent (first-order) regularities (for a review see Wilson et al., 2020), we investigated the effect of temporal spacing in temporal category learning a Type I regularity in Study 1. However, we found no conclusive evidence for or against a corresponding effect on learning and generalization. While this seems not entirely in line with research on artificial grammar-learning (Newport et al., 2004; Gomez, 2002) and in the serial reaction task paradigm (Lazartigues et al., 2022), we highlighted above that also in artificial grammar-learning adjacency effects seem moderated by factors that can affect how perceptually close the related elements are perceived (see Wilson et al., 2020). In our study, this could concern the positioning of the relevant elements at the beginning and the end of the non-adjacent sequence, or that the irrelevant task was presented in a different format (estimation instead of categorization). In this vein, categories have been argued to be more salient than stimulus features (or values) in category learning (e.g., Deng & Sloutsky, 2012; Johansen et al., 2015), which may have facilitated noticing the regularities between the two categorization tasks in Study 1. However, the latter possibility would also imply that this might be the case in both adjacent and non-adjacent conditions, and that the remaining influence of temporal distance (e.g., on maintaining the relevant information) was rather small. In this vein, we believe that it is fruitful to study whether performance further depends on positioning (e.g., adjacent with O$^2$ predicting O$^3$), or whether the behavioral patterns change if the irrelevant task in Study 1 was also a categorization task, which warrants further investigation.

More specifically, while we found an omnibus interaction regarding task and condition (adjacent vs. nonadjacent) in Study 1, this was mostly due to a within-condition benefit of the target task in the adjacent condition (quicker learning of O$^2$ compared to O$^1$), which was not observed in the non-adjacent condition. However, the between-condition differences on the target task O$^2$ between both conditions remained inconclusive, and studies reporting corresponding effects in the artificial grammar-learning domain seem to rely on within-participant comparisons as well (e.g., Deocampo et al., 2019). Thus, to also find reliable between-condition effects in direct comparison might warrant larger sample sizes in future studies. However, regarding subsequent Type I generalization, the evidence favored the absence of an effect between adjacent and non-adjacent condition, which could further highlight a general aspect of the sensitivity of the test and its potential to increase the salience towards a regularity in post-hoc reasoning (i.e., after learning: see also Frensch & Rünger, 2003). That is, in our test phase, we introduced novel stimuli for which no competing memory traces existed. This may have led participants to consider how to predict categories without feedback, potentially facilitating retrospective recognition of the regularity. This, in turn, may have allowed them to use it to categorize the novel stimuli equally in both adjacency conditions. In other words, in Study 1, this process could have equalized the generalization trends (i.e., compensated for a weaker recognition of the regularity in the non-adjacent condition).

### Limitation and future directions

While bringing together both category and temporal regularity, the current design extension to investigate Type I and Type II generalization in a transfer phase in our studies provides an interesting novel test-bed for theories focusing on classical visual category learning structures. That is, the classic six problems are typically only studied regarding training performance (e.g., Shepard et al., 1961; Lewandowsky, 2011; Nosofsky et al., 1994a), because the stimuli consist of three binary features exhausting the stimulus space during training. One main advantage of learning inter-category relations in successive decisions, for example, our temporal Type I problem, is that it easily allows inspecting generalization trends beyond the training stimuli, since the visual stimuli could be easily replaced (or removed). Typically, however, category learning models do not make an explicit distinction between learning and generalization (see Pothos & Wills, 2011), but assume that learning benefits arise, in fact, *due to* generalization (i.e., abstracting away from irrelevant predictors), for which the current task design could provide insightful evidence in future applications (e.g., by manipulating factors at test). In this regard, we believe that also investigating how pairing both predictive visual and sequential information (e.g., either in competition or being redundant) could further elucidate how people learn and generalize causal relations in future studies.

Besides the possible theoretical implications, there are also some limitations. As mentioned above, unlike Study 1, which was conducted in a controlled lab environment, Study 2 was conducted online on Prolific. As expected in a more diverse sample, which could lower performance in general (see. Dandurand et al., 2008; Birnbaum, 2004). However, to ensure data quality, we included control questions to verify that the instructions had been understood, and applying performance-based exclusion criteria did not change the main conclusions. One aspect that limits comparisons to other domains, such as artificial grammar-learning, is that, in our studies, we focused on accuracy as a measure of learning performance, while other studies base their inferences on differences in reaction times (e.g., in anticipation of a predictable response). We further did not collect verbal protocols or survey questions designed to measure the participants’ insights into the corresponding rule solutions. Given the prominence of the question of implicit versus explicit learning in the temporal learning literature (see Frensch & Rünger, 2003) as well as the potential importance of prior knowledge in shaping which regularities are detected, using verbal protocols to investigate participants’ strategies and insight seems fruitful for future studies.

Finally, as highlighted in the previous section, introducing different visual stimuli might reveal some boundary condition for the observed effects. Here, we used abstract idiosyncratic symbols as initial visual stimuli (e.g., a dollar sign) preceding an associated sequence of decisions, which could be memorized as such altogether. On the one hand, using less abstract or less idiosyncratic symbols might lead to different learning strategies, for example, if the initial visual stimuli themselves included systematic feature dimensions (e.g., color or size), as typical in visual category learning. Participants could try using selective features to predict successive outcomes (e.g., blue leads to A, green to B of the second categorization in the sequence). Indeed, it seems more realistic that both visual predictors might compete with other predictors for attention for predicting correct responses, including sequentially presented information as in our task. It remains an open question whether the corresponding Type I inter-category relations would still be learned if those visual stimuli were diagnostic, warranting further research.

## Conclusion

Our research examined whether people can learn correlations between multiple categories when making a series of decisions about the same stimulus. This goes beyond traditional category learning tasks, focusing on stimulus-category relations. We compared conditions with existing temporal regularities between these categories with conditions in which only configural-memory-based learning was possible. We found that people are sensitive to these regularities. Further learning depended on the complexity of the structure, while temporal proximity between the correlated category-outcomes was less important. In particular, people were able to generalize rules in the simple Type I structure, but not in the more complex Type II (disjunctive) structure. This differs from visual categorization studies, where people usually learn both rule types more easily than unstructured problems. We believe that these insights open up fruitful new avenues and research questions when combining methods from visual category learning and artificial grammar-learning. We speculate that factors such as cognitive load and prior knowledge might further moderate these influences, warranting further research.

## Supplementary Information

Below is the link to the electronic supplementary material.

Supplementary file 1 (pdf 152 KB)

## Data Availability

All the data, analysis files, and experimental setup files are available at https://osf.io/ymu4b/?view_only=b05942441669406eb04673453094a9ee. The link is also available with other pre-registration links in the manuscript.

## Footnotes

1 The overall pattern of results remains the same when applying EM1 or EM2 exclusions except that, in both, we found a significant main effect for condition indicating better performance in the adjacent condition, $\chi ^{2}$(1, 70) = 5.39, *p* = .02. In addition we found a significant interaction between blocks and condition suggesting faster learning in the adjacent than the control condition, $\chi ^{2}$(1, 70) =7.20, *p* = .007. The differences occurred independent of the exclusion method. Thus, the reported values are based on the EM2 model.

2 When applying the exclusions, we additionally found a significant 3-way interaction between blocks, decision task, and condition with both exclusion methods EM1 and EM2, suggesting that learning speed depended on condition and decision tasks, EM2: $\chi ^{2}$(1, 70) =6.21, *p* = .013.

3 Regarding reaction times, we found anecdotal evidence for the absence of a difference between the adjacent and the control condition, $BF{10} =0.31$, when applying exclusion method EM1 (similar for EM2). Regarding accuracy in O$^2$, we further found a significant difference of condition $X^{2}$(1, 81) =8.68, *p* = .013, with higher accuracy in the adjacent condition compared to control. Thus, these results are inconsistent with the idea of easier recall of S$\rightarrow$O$^2$ associations.

4 All research materials, including datasets, analysis scripts, and experiment files, are available on the Open Science Framework (OSF) repository at https://osf.io/ymu4b/?view_only=b05942441669406eb04673453094a9ee

5 When excluding participants using the EM1 model, we find the same pattern of results, with the exception of a significant interaction between blocks and tasks, $X^{2}$(1, 66) = 8.70, *p* = .013. which further indicated quicker learning in O$^1$ and the slowest learning in O$^2$

6 One difference in these results emerged when applying the EM1 exclusion method resulting in a significant interaction of blocks and decision task suggesting difference in learning accuracy in each decision task based on blocks, ($\chi ^{2}$ (1, 66) = 6.38, *p* = .041 in the training phase. The central conclusions of our hypotheses, however, remained the same.
