# Supplementary material for: Learning Type I and Type II regularities between multiple sequentially presented stimulus categories
Source: Psychol Res. 2025 Sep 18;89(5):144. doi: 10.1007/s00426-025-02180-7 (PMC12446112; doi:10.1007/s00426-025-02180-7)
Supplement: Supplementary file 1 — (pdf 152 KB) [file 426_2025_2180_MOESM1_ESM.pdf]

Supplementary Document

S1

Study 1: Test phase accuracy of Old targets

In the test phase, we first hypothesized that participants categorize the *previously trained* (old) stimuli equally well or more accurately on O^2 in the adjacent regularity condition, compared to the control condition. Figure 1 depicts the main results, suggesting a corresponding trend. To test this, we performed the same mixed-effects logistic regression model as the one performed for the training phase. Still, to avoid singularity issues, we reduced the model with by-participant and by-stimulus random intercepts and by-participant random slopes for the main effects of decision tasks, focusing on the four previously trained stimuli. We found a significant main effect of blocks,($X^2(1, 70) = 9.73$, $p = .002$, and an interaction effect between decision task and condition,$X^2(1, 70) = 8.29$, $p = .004$, as well as between decision task and blocks, $X^2(1, 70) = 15.01$, $p < .001$. There was no significant main effect of condition, $X^2(1, 70) = 3.02$, $p = .08$, and decision task, $X^2(1, 70) = 0.98$, $p = .32$, and no interaction between condition and blocks ($X^2(1, 70) = 0.04$, $p = .835$, or a three-way interaction, $X^2(1, 70) = 0.23$, $p = .629$. The interaction between decision task and block is unsurprising as learning continued with O^1 feedback, while feedback was absent for O^2. Regarding the latter, participants performed at approximately the same level as in the final training blocks.

In this vein, a post-hoc analysis on the interaction between decision task and condition revealed that O^2 accuracy was reliably higher in the adjacent condition compared to the control, *Mdiff* = 1.32, $p = .007$, *CI95*= [0.35,2.29], $z = 2.68$. Furthermore, the mean accuracy was not higher on O^2 compared to O^1 within the adjacent condition *Mdiff* = -0.33, $p = .24$,*CI95* = [-0.88,0.22], $z = -1.17$, however it was lower on O^2 than in O^1 within the control condition, *Mdiff* = 0.76, $p = .003$, *CI95* = [0.26,1.26], $z = 2.96$. As for the previous training phase, there was no difference in accuracy in O^1 between conditions, *Mdiff* = 0.24, $p = .60$, *CI95*= [-0.66,1.13],$z = 0.52$. Generally, thus, the results align with those from the training phase, indicating better performance on O^2

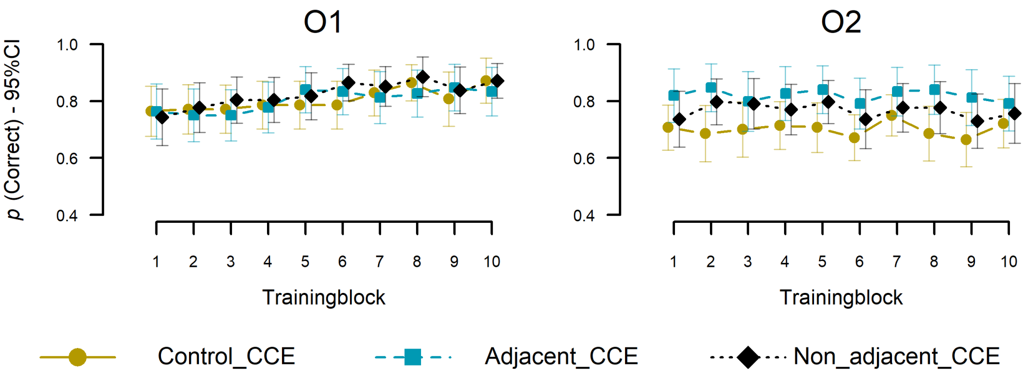

Figure 1

Test phase mean accuracy (old targets). Accuracy (y-axes) over training blocks (x-axes; 8 trials each block) for C1 categorizations (left) and C2 (right). Error bars indicate 95% CIs of individual means.

with adjacent regularity compared to the control condition. [1]

Regarding the comparison between the adjacent and non-adjacent Type I conditions, we conducted the same tests revealing a significant main effect of blocks, $X^2(1, 72) = 10.59$, $p = .001$, and between decision task and blocks, $X^2(1, 72) = 21.64$, $p < .001$, as well as conditions and decision task, $X^2(1, 72) = 4.02$, $p = .045$, reflecting the similar patterns as for the comparison between adjacent and control condition. Again, we neither found a significant main effect of condition, $X^2(1, 72) = 0.40$, $p = .53$, nor the decision task, $X^2(1, 72) = 0.14$, $p = .71$, and no interaction between condition and block, $X^2(1, 72) = 0.21$, $p = .648$, or a three-way interaction, $X^2(1, 72) = 0.19$, $p = .661$.

Post-hoc analyses further confirmed that the mean O^2 accuracy was not higher in the adjacent

[1] We found significance in the main effect condition after excluding participants based on both exclusion models. According to the EM1, we have significance in main effect condition $\chi^2(1, 70)$ =8.88, p =.003, suggesting higher overall accuracy in adjacent condition compared to control. A posthoc test indicated higher accuracy in O^1 in adjacent condition compared to control condition *Mdiff* = 1.01, p = .046, *CI95*= [0.018,1.99], z = 3.50, which clearly would result in higher accuracy in adjacent condition overall. This could be the result of higher performing participants based on O^1

compared to the non-adjacent condition, $Mdiff$ = 0.67, p = .22 , $CI95$ = [-0.41,1.76], z = 1.21, as well having no difference the O^1, $Mdiff$ = -0.06, p = .90, $CI95$ = [-0.97,0.85], z = -0.13. Also, no accuracy difference for O^2 compared to O^1 in the non-adjacent condition, $Mdiff$ = 0.29, $p = .26$, $CI95 = [-0.99, 0.1]$, z = 1.17. Importantly, these overall results merely reflect the same trends as during prior learning, and in the next analyses, we turn to the question of how participants responded to novel targets concerning whether or not participants represented a category-category regularity beyond idiosyncratic stimulus-category associations. [2]

S2

Study 1 - Explorative Analyses: Position effect

The training phase above showed no learning difference in the adjacent compared to the non-adjacent condition except when the exclusion model (EM1) filtered out low-performing participants, resulting in a learning difference. At the same time, we did not find differences in using the regularity for generalization to novel stimuli. A potential alternative explanation for the effects was position effects: In the adjacent condition, the categorization task predicted by the regularity was the second task, and in the non-adjacent, the third task. Thus, to investigate whether position affected the ease with which the tasks were learned we analyzed the performance in the estimation task (filler task) which appeared on position three in the adjacent condition ($Mean = 49.8$, $Sd = 21.7$) and control condition ($Mean = 47.9$, $Sd = 18.5$) and on position two in the non-adjacent condition($Mean = 45.60$, $Sd = 19.7$). We performed a mixed-effects logistic regression model on the accuracy in the estimation task (1= correct; 0 = incorrect) with blocks and condition as fixed effects (the latter now reflecting position 2 vs. position 3) and by-stimulus random intercepts and with random slopes with blocks nested in by-participant random intercepts. We found a significant main effect of training blocks (X^2 (1, 107) = 148.37, $p < .001$), no main effect of condition (X^2 (1, 106) = 0.84, $p = 0.658$), but no

[2] We do not find a significant interaction between condition and task, X^2(1, 72) = 3.41, $p = .065$, after filtering participants based on both the exclusion model. Note: the result of non-significance is reported based on participants after EM1 exclusion model

interaction between blocks and conditions (X^2 (1, 106) = 1.09, p = 0.58). This would suggest no difference in accuracy based on condition and no difference in learning in the condition over blocks. As there was no reported difference in accuracy between adjacent and non-adjacent conditions post-testing, rendering position effects an unlikely alternative cause of this effect.

S3

Study 2: Testing Accuracy of Old targets

We hypothesized that *previously trained* (old) stimuli were categorized equally well or better for task 3 in the Type II condition compared to the control condition based on the hypothesis that Type II learning be acquired in the training phase. Figure 2 depicts the main results, again suggesting equal performance between tasks and conditions, as during previous training. We performed a mixed-effects logistics regression model like in the training phase, focusing only on the four previously trained stimuli but had to use a reduced version to avoid overfitting and singularity issues, with blocks and conditions as main effects and by-participants and by-stimulus as random intercepts and decision task as the random slope in by-participant intercepts in order to account for learning difference in task sequence. We found a significant main effect of decision task, X^2(1, 66) = 6.01, p = .049, suggesting an overall accuracy difference between the tasks but no significance in blocks,(X^2(1, 67) = 0.07, p = .791, and condition X^2(1, 67) = 0.48, p .487. There was no significant interaction between condition and blocks (X^2(1, 67) = 0.36, p = .55, or between decision task and blocks (X^2(1, 66) = .91, p = .634 or between decision task and condition (X^2(1, 66) =1.44, p = .49 or a three-way interaction, X^2(1, 66) = 1.24, p = .54.

We additionally performed a post-hoc analysis on the interaction between decision task and condition (as pre-registered) that further confirmed that there is no difference in O^3 accuracy in the Type II condition compared to the control condition, *Mdiff* = 0.32,p = .40, *CI95* = [-0.43,1.07], z = 0.84. Thus, the test phase mainly replicates the previous results from the training phase, showing no differences in O^3 accuracy. [3]

[3] After excluding participants based on EM2 model, we encountered no significance in decision task X^2(1, 66) = 5.58, p = .06, negating any between task differences

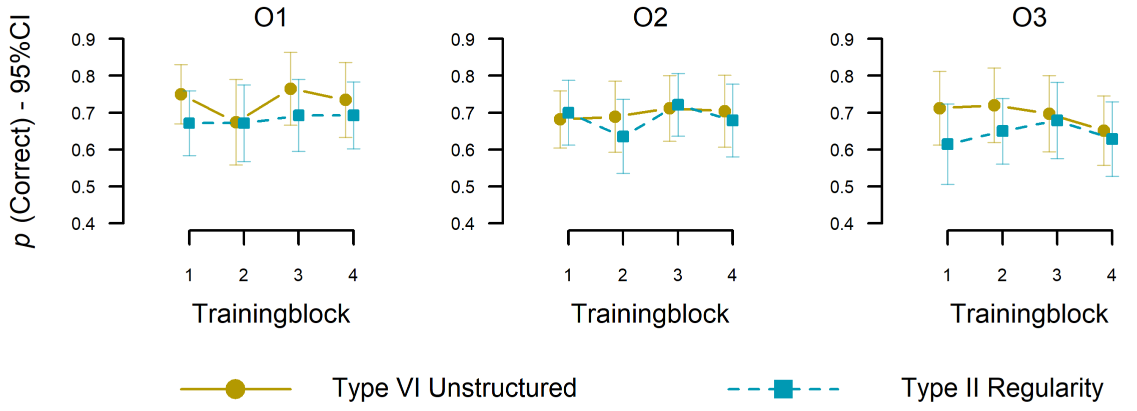

Figure 2

Test phase mean accuracy (old targets). Accuracy (y-axes) over testing blocks (x-axes; 4 trials each block) for O1 categorizations (left), O2 (middle) and O3 (left). Error bars indicate 95% CIs of individual means.
